# Poster 1007: Universal allergen particle generation for inhaled allergen challenges in the fraunhofer environmental challenge chamber (ECC)

**DOI:** 10.1186/1939-4551-7-S1-P4

**Published:** 2014-02-03

**Authors:** Jens M Hohlfeld, Katrin Lueer, Heike Biller, Horst Windt, Philipp Badorrek, Meike Mueller, Holger Ziehr, Dietrich Haefner, Norbert Krug, Wolfgang Koch

**Affiliations:** 1 Fraunhofer Institute, Hannover, Germany; 2 Allergopharma GmbH & Co KG, Reinbek, Germany

## Background

Efficacy testing of immunotherapy in field studies is often hampered by variation of airborne allergens. Standardized allergen exposure in challenge chamber settings might overcome aforementioned limitations. However, airborne allergens such as house dust mite (HDM) or cat allergen are not available as standardized source material with distinct particle size. Therefore, we developed a method for universal allergen particle generation and conducted a pilot study to clinically validate this challenge paradigm for house dust mite.

## Methods

House dust mite allergen extract (ALK lyophilised SQ503 Der p, ALK-Abello, Wedel, Germany) was diluted in an aqueous solution of 10% lactose. The solution was spray-dried at various liquid feed rates leading to allergen aerosols with different pre-defined concentrations but a constant mass mean aerodynamic particle diameter of 13.5 µm. Particle size is dependent on the solute (lactose) concentration and can thereby be adjusted accordingly. In a single-blind, five-way cross-over pilot study 18 subjects with allergic rhinitis and sensitization to HDM were allergen-exposed for 4 hours at either 250 SQE/m^3^, 500 SQE/m^3^, 1000 SQE/m^3^, or lactose alone (0 SQE/m^3^) seven days apart. The dose of 500 SQE/m^3^ was repeated to investigate reproducibility. Total nasal symptom score (TNSS), anterior rhinomanometry, nasal secretions, exhaled NO, FEV1, and adverse events were assessed prior to and during the exposures.

## Results

Allergen exposure was safe and significantly elicited symptoms of AR compared to room air exposure with a mean total nasal symptom score (TNSS) of 3.6±2.0 (mean±SD) at the highest allergen concentration. Lactose alone did not change TNSS (0.7±0.6) compared to pre-challenge level. Repeated exposure to 500 SQE/m^3^ induced a TNSS which was not different between the two challenge sessions. Objective measures of nasal flow and nasal secretions were in line with clinical symptoms.

## Conclusions

We conclude that this universal allergen particle generation is safe, specific, and reproducible and can therefore be used for efficacy testing of immunotherapy.

